# Acute cerebellar ischaemic stroke secondary to arterial thoracic outlet syndrome

**DOI:** 10.1093/jscr/rjac296

**Published:** 2022-06-24

**Authors:** Balamrit Singh Sokhal, Leila Mohammadi, Janaka Weerathunga, Sriram Rajagopalan

**Affiliations:** School of Medicine, Keele University, Keele, Staffordshire, UK; School of Medicine, Keele University, Keele, Staffordshire, UK; University Hospital North Midlands, Stoke-On-Trent, Staffordshire, UK; University Hospital North Midlands, Stoke-On-Trent, Staffordshire, UK

**Keywords:** Stroke, Cervical rib, Thoracic outlet syndrome, Vascular surgery

## Abstract

Arterial thoracic outlet syndrome comprises a collection of symptoms due to compression of the neurovascular structures of the thoracic outlet. Cervical ribs are rare congenital abnormalities that are a cause of thoracic outlet syndrome, leading to upper limb complications depending on the compressed structure. Management tends to be surgical in the form of rib resection. We report a case of arterial thoracic outlet syndrome secondary to a cervical rib in a 45-year-old male who presented with left-sided cerebellar stroke caused by subclavian artery thrombosis. Medical management in the form of anticoagulation was favoured. Oedema in the recent peri-infarct zone due to reperfusion may have caused compression of the fourth ventricle due to anatomical proximity, with the risk of further neurological compromise and coning. At follow-up appointments, the patient had no residual upper limb or neurological symptoms.

## INTRODUCTION

Cervical ribs (CRs) are rare congenital anomalies that occur in <1% of the population and are found to be the underlying aetiology in 29% of individuals suffering from thoracic outlet syndrome (TOS) [1]. TOS comprises various conditions that cause compression of the neurovascular components of the thoracic outlet. CRs are a cause of arterial thoracic outlet syndrome (A-TOS), the rarest form of TOS caused by compression of the subclavian artery [2]. Symptomatic A-TOS is rare and often presents with upper limb ischaemic symptoms due to antegrade thrombo-embolization. Retrograde thrombo-embolic events are rarer but have been reported to cause transient ischemic attack (TIA) and stroke [3]. We report a case of CR and A-TOS in a 45-year-old male who presented with left-sided cerebellar stroke caused by subclavian artery thrombosis.

## CASE REPORT

A 45-year-old male attended his local accident and emergency department with a 1-day history of dizziness, unsteadiness and episodes of vomiting. This was alongside a 4-month history of worsening claudication in the left arm. On neurological examination, he was noted to have nystagmus, vertigo, ataxia and left eye movement lag on lateral gaze, arising suspicion of stroke. Index vascular examination demonstrated a relatively cool left upper limb, forearm to hand with mild hand grip deficit, associated with absent brachial, radial, and ulnar pulses and a delayed capillary refill time. There was no rest pain or tissue loss. The patient had no other relevant history.

**Figure 1:**
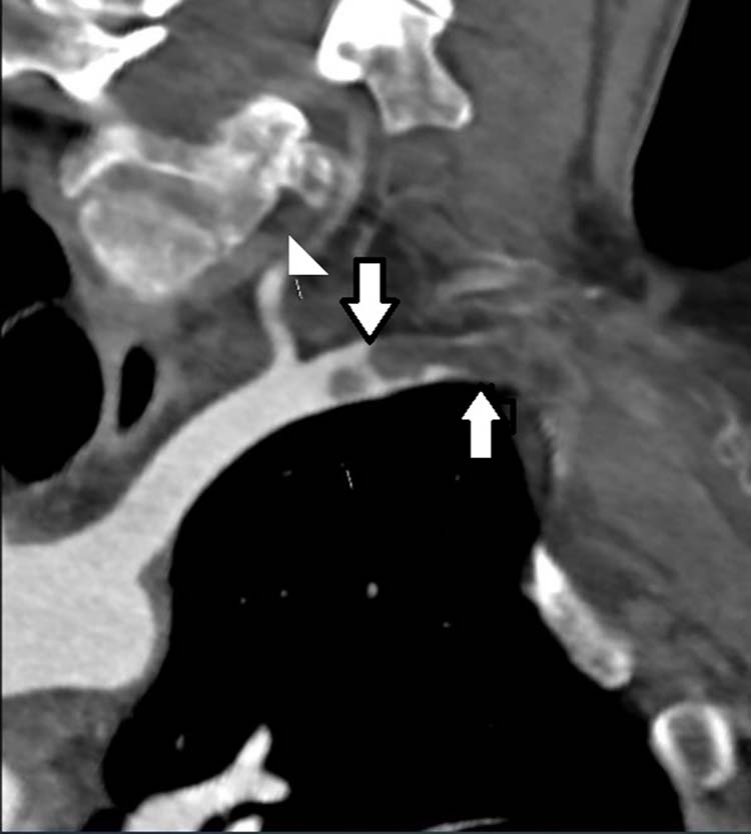
CT angiogram demonstrating location of the thoracic outlet obstruction (up arrow) and retrograde thrombosis (down arrow).

Electrocardiogram showed normal sinus rhythm. The patient’s sudden onset dizziness was investigated with a non-contrast computed tomography (CT) head and was negative for an acute bleed. A CT angiogram of the aortic arch, both common carotids and both upper limbs was performed, which confirmed subclavian artery thrombosis secondary to a left-sided CR and occlusion of subclavian artery, with collateral forearm vessels (Fig. 1).

**Figure 2:**
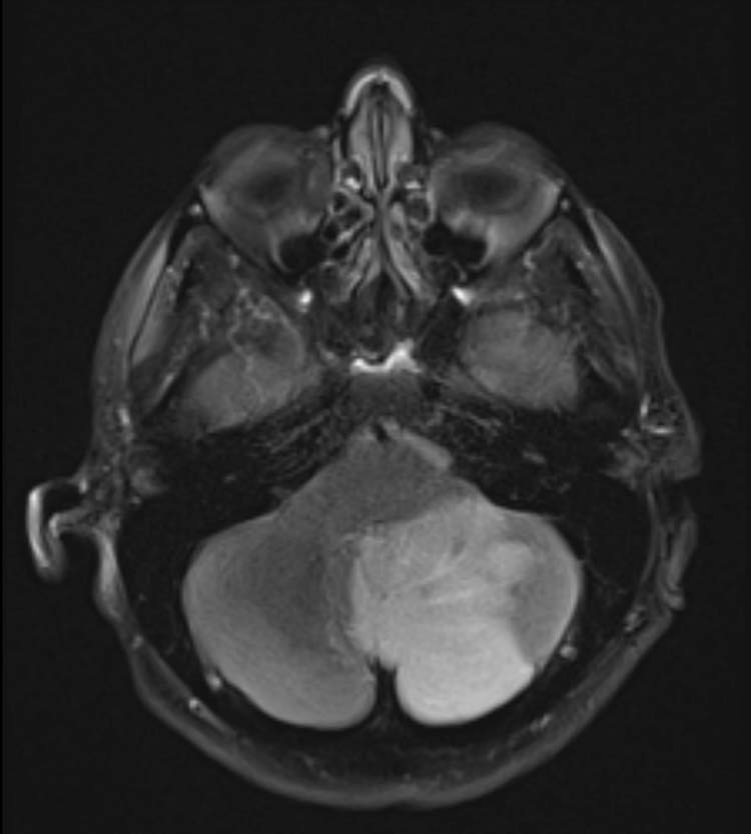
T2 sequence magnetic resonance imaging demonstrating large left cerebellar ischaemic stroke.

The patient was given an intravenous heparin bolus and transferred to a tertiary vascular centre. At this point, his posterior circulation stroke was not confirmed on imaging although suspected clinically. On review by the vascular team, the patient reported that his left arm symptoms were improving since heparinization. Motor skills, sensation, capillary refill and colour in the limb progressively improved. However, due to persistent dizziness, a magnetic resonance imaging head was performed, which confirmed a large acute left cerebellar infarct (Fig. 2).

After careful consideration and discussion with the patient regarding surgical resection of the rib and arterial revascularization, medical management was the preferred option. Haemodynamic fluctuations due to anaesthesia and oedema in the recent peri-infarct zone due to reperfusion may have caused compression of the fourth ventricle due to anatomical proximity, with the risk of further neurological compromise and potential coning. There was also an increased risk of bleeding in the peri-infarct tissue during or immediately post-surgery, which could also lead to coning.

The patient was treated with long-term anticoagulation for his ischaemic stroke and ischaemic limb and then changed to a new oral anticoagulant (Apixaban). They were repatriated to the local hospital for rehabilitation and reviewed in the vascular clinic after 2 and 6 months. Two months on from his initial presentation, his arm was completely asymptomatic. After 6 months, the patient opted against rib resection and bypass surgery.

## DISCUSSION

CRs are rare congenital anatomical abnormalities seen in <1% of the population and are often asymptomatic [1]. CRs are a cause of TOS, accounting for 29% of all TOS cases [2]. TOS is a group of conditions that relate to compression of the neurovascular bundle of the thoracic outlet, namely the brachial plexus and the subclavian artery and vein [1]. Arterial thoracic outlet syndrome (ATOS) is the rarest form of TOS caused by compression of the subclavian artery which can be secondary to a CR [2]. ATOS is usually asymptomatic until an embolic event occurs [3, 4]. Compression and subsequent obstruction of the subclavian artery or a post-stenotic dilatation of the artery can cause anterograde thrombo-embolic events leading to upper limb ischaemic symptoms including paraesthesia, pain, pallor, coldness, absent pulses and prolonged capillary refill time. Retrograde thrombo-embolic events are rarer but have been reported to present as TIA or stroke [3, 5].

The mechanism by which A-TOS causes stroke is poorly understood. A review of 33 patients with ischaemic stroke associated with A-TOS found that 26 had a CR. Twenty-one of these patients demonstrated ATOS symptoms prior to the stroke with only a minority being diagnosed with TOS before the stroke. The median age was 21 years and 50% were male and symptoms ranged from claudication to limb ischaemia. All had absent peripheral pulses. Twenty patients with a CR went on to have imaging, where 8 demonstrated occlusion at the axillary artery and beyond. Four patients were reported to have posterior circulation infarcts, with only one case due to left-sided subclavian artery occlusion [5].

Our patient reflected aforementioned study. They solely reported a 4-month history of left upper limb claudication symptoms. We now know these symptoms were likely due to the compression of the subclavian artery by a CR. Four months later, he presented with symptoms of posterior circulation stroke. He was a non-smoker with no known cardiac illness, or risk factors for atherosclerosis. We propose that mechanical compression of the subclavian artery led to damage of the tunica intima and thrombus formation [5]. His arm deterioration could be a result of complete thrombotic occlusion of a previous stenotic artery. His ataxia was due to retrograde extension of thrombosis to involve vertebral artery origin and antegrade embolization to distal vertebral artery and posterior circulation. His limb was likely to have collateral blood supply due to insidious claudication and hence not present as an acute limb ischaemia. Therefore, this scenario prompts any patient presenting with posterior circulation ischaemic stroke with a history of upper limb claudication to be investigated for A-TOS, and hence, the importance of a detailed history and examination to guide correct investigations cannot be understated.

## CONFLICT OF INTEREST STATEMENT

None declared.

## FUNDING

None.
